# Impact of *Scutellonema curcumae* sp. n. (Nematoda: Hoplolaimidae) on the Phytochemical Profile and Biological Activities of Turmeric (*Curcuma longa* L.)

**DOI:** 10.3390/molecules31060920

**Published:** 2026-03-10

**Authors:** Tu Thi Dinh, Quan Minh Pham, Long Quoc Pham, Chi Kim Ngo, Van Thi Thuy Nguyen, Thuong Thi Le Hoang, Tu Ngoc Ly, Linh Ngoc Nguyen, Thao Thi Phuong Nguyen, Lam Tien Do

**Affiliations:** 1 Faculty of Chemistry, Graduate University of Science and Technology, Vietnam Academy of Sciences and Technology, 18 Hoang Quoc Viet, Nghia Do, Hanoi 100000, Vietnam; 2 Institute of Chemistry, Vietnam Academy of Sciences and Technology, 18 Hoang Quoc Viet, Nghia Do, Hanoi 100000, Vietnam; 3 Laboratory of Biophysics, Institute for Advanced Study in Technology, Ton Duc Thang University, Ho Chi Minh 700000, Vietnam; 4 Faculty of Pharmacy, Ton Duc Thang University, Ho Chi Minh 700000, Vietnam; 5 Department of Forensic Science, People’s Police Academy, Co Nhue 2, Bac Tu Liem, Hanoi 100000, Vietnam; 6 Faculty of Education, Tan Trao University, Tuyen Quang 220000, Vietnam; 7 Graduate Center of Sciences and Technology, Hanoi Metropolitan University, 98 Duong Quang Ham, Nghia Do, Hanoi 100000, Vietnam; 8 Institute of Medicine and Pharmacy, Thanh Do University, Lai Xa, Hanoi 100000, Vietnam; 9 Institute of Biological and Food Technology, Hanoi Open University, B101 Nguyen Hien, Bach Mai, Hanoi 100000, Vietnam

**Keywords:** Kon Tum, taxonomy, *Scutellonema*, 28S, molecular, turmeric, curcuminoid, bisdemethoxycurcumin

## Abstract

A new spiral nematode species, *Scutellonema curcumae* sp. n., was identified from the rhizosphere of turmeric (*Curcuma longa* L.) in the Western Highlands of Vietnam. Integrative taxonomical analysis, combining detailed morphology and molecular characterization (ITS, 28S D2–D3 rDNA, and COI mtDNA), confirmed its distinctiveness. *Scutellonema curcumae* sp. n. is characterized by a unique combination of a spiral body, a hemispherical lip region with four annuli, a robust stylet, and a rounded tail with a prominent scutellum, forming a highly divergent lineage within the genus. Beyond its description, this study reveals a significant inverse correlation between nematode population density and the phytochemical quality of the host. High infestation levels were associated with a marked decline in total curcuminoid content. Notably, lower nematode density favored a specific shift in the curcuminoid profile, with bisdemethoxycurcumin levels increasing by up to 250%. These phytochemical alterations directly influenced the therapeutic potential of the rhizomes: lower infestation levels resulted in significantly enhanced antioxidant capacity (lower SC_50_ values) and cytotoxic activity (lower IC_50_ against HepG2 and A549 cell lines). This work represents the first report of a *Scutellonema* species associated with turmeric in Vietnam and underscores its detrimental impact on the medicinal and nutraceutical value of the crop. Our findings suggest that effective nematode management is crucial not only for yield protection but as a strategic intervention in precision agriculture to optimize the secondary metabolite profiles of medicinal plants.

## 1. Introduction

The genus *Scutellonema* Andrássy, 1958 comprises a group of spiral nematodes that are widely distributed in tropical and subtropical regions, where they are commonly associated with the roots of a broad range of cultivated and wild plants [1,2].

Since its establishment by Andrássy [3], the genus *Scutellonema* has expanded significantly. Following the comprehensive revision by [4,5] and subsequent descriptions [6,7,8,9], approximately 50 valid species are currently recognized worldwide, distinguishable primarily by the structure of the lip region, scutella size, and reproductive morphology.

Although most *Scutellonema* spp. are not regarded as major crop pests, several species, particularly *S. bradys*, *S. cavenessi*, and *S. brachyurum* are known to cause significant economic losses in a range of crops such as yam, cotton, soybean, peanut, maize, and sugarcane [1]. Feeding by these nematodes disrupts root epidermal and cortical tissues, leading to impaired nutrient and water uptake, stunting, leaf chlorosis, and reduced yield. The extent of damage varies among species, but economic consequences can be substantial in systems where nematode pressure is high or where crops are cultivated continuously [1,2].

Species identification within the genus remains challenging due to morphological conservatism and overlapping diagnostic characters, such as lip region configuration, structure of the reproductive system, position of the hemizonid and secretory–excretory pore, and size and shape of the scutella [4,10]. Distinguishing closely related taxa, such as those within the *S. bradys* complex, has proven particularly difficult [6,9]. As a result, integrative approaches combining morphological examination with molecular tools (e.g., 28S D2–D3, ITS, COI) have become essential for accurate species delimitation and phylogenetic inference [6,9].

In Vietnam, *Scutellonema* represents a relatively diverse genus, with ten species currently reported: *S. amabile*, *S. brachyurum*, *S. brevistyletum*, *S. dentivaginum*, *S. hoabinhensis*, *S. paramovovi*, *S. sheri* (syn. *S. brachyurum*), *S. siamense*, *S. tanlamense*, and *S. vietnamense* [8,11]. Data regarding their association with medicinal plants remains scarce. However, previous reports of *Scutellonema spp.* from Vietnam were based solely on limited morphological observations without accompanying molecular data, leaving uncertainties regarding the identity and diversity of *Scutellonema* populations in the country.

Turmeric (*C. longa* L.) is a plant of substantial economic and medicinal importance. It is widely used as a culinary spice and as a raw material in traditional and modern medicine due to its abundance of pharmacologically active compounds, including curcuminoids, essential oils, and phenolics. These compounds contribute to a wide spectrum of biological activities, such as anti-inflammatory, antioxidant, antidiabetic, hepatoprotective, anticancer, and antimicrobial effects [12,13]. Despite its importance, turmeric is susceptible to a variety of plant-parasitic nematodes. Global reports show that *Meloidogyne incognita*, *M. javanica*, *Rotylenchulus reniformis*, *Pratylenchus* spp., and *Radopholus* spp. can cause severe damage to turmeric roots and rhizomes [1,14]. Nematode infestations frequently result in root galling, cortical necrosis, rhizome rot, plant stunting, and considerable yield reduction. Moreover, nematode infection has been shown to alter the physiological and biochemical pathways of turmeric. Several studies have demonstrated that nematode-induced stress can reduce rhizome biomass and significantly lower concentrations of key bioactive constituents such as curcumin, demethoxycurcumin, and bisdemethoxycurcumin—compounds central to the medicinal quality and economic value of turmeric [15,16]. Some nematodes, such as *Meloidogyne* spp., could disrupt the production of multiple classes of plant metabolites, including phenolics, alkaloids, amino acids, and carbohydrates, which are often associated with plant defense responses [17]. Such nematode-induced metabolic perturbations may consequently modify essential oil composition and reduce overall curcuminoid content, thereby diminishing both the therapeutic properties and market quality of harvested rhizomes. These effects emphasize the necessity of early detection and efficient nematode management in turmeric production systems.

In Vietnam, research on nematodes associated with turmeric remains limited. Only *Rotylenchulus reniformis*, *Meloidogyne incognita*, and *M. javanica* have been reported from turmeric fields [11], and no studies have documented the presence of *Scutellonema* spp. or other spiral nematodes on this crop. The absence of baseline data represents a major gap in the development of integrated pest management strategies for turmeric in the region.

During a recent nematological survey in the Western Highlands of Vietnam, a population of *Scutellonema* was recovered from the rhizosphere of turmeric. Detailed morphological and molecular analyses confirmed that this population represents a new species, herein described as *Scutellonema curcumae* sp. n. In this study, we also analyze the relationship between nematode infection and turmeric’s chemical composition to assess potential effects on key bioactive compounds. The objectives of the present study were therefore to: (i) provide a comprehensive morphological and molecular characterization of *Scutellonema curcumae* sp. n.; (ii) compare it with closely related congeners; (iii) infer its phylogenetic position within the genus; and (iv) evaluate its association with chemical constituents and biological activity of turmeric. This work contributes to the growing understanding of nematode biodiversity in Southeast Asia and highlights the importance of identifying nematode pests that may reduce both yield and chemical quality in high-value medicinal crops such as turmeric.

## 2. Results

### 2.1. Scutellonema curcumae sp. n.

**Systematic**

Order Rhabditida Chitwood, 1933.

Family Hoplolaimidae Filip’ev, 1934.

**Figure 1:**
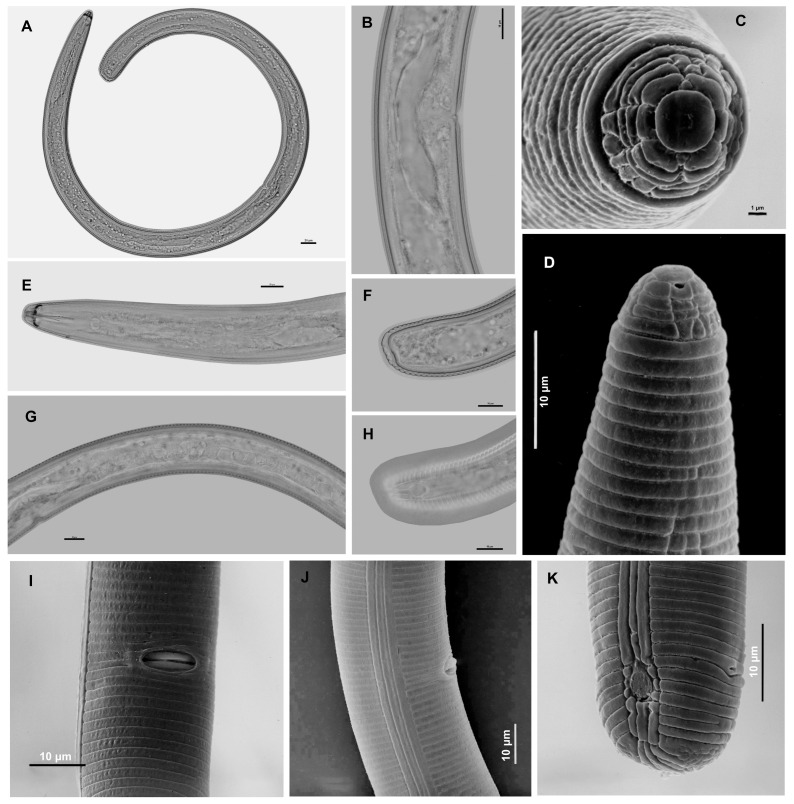
Females of *Scutellonema curcumae* sp. n. from Vietnam. (**A**): Entire body; (**B**,**I**,**J**): Vulva region; (**C**,**D**,**E**): Anterior region; (**F**): Tail region; (**H**,**K**): Tail region showing scutella; (**G**): Ovary. (Scale: (**A**): 20 µm; (**B**,**D**–**K**): 10 µm; (**C**): 1 µm).

**Figure 2:**
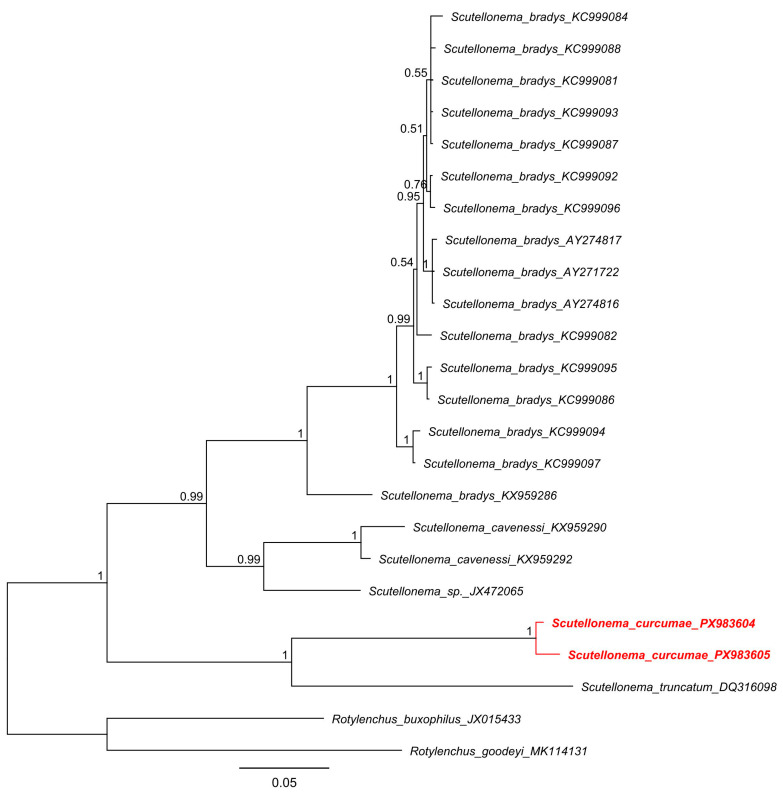
Bayesian phylogenetic tree generated using ITS sequences of *Scutellonema* species under the GTR + G + I model. The sequence of *Scutellonema curcumae* sp. n. from Vietnam was indicated by bold font and red color.

Species *Scutellonema curcumae* sp. n.

(Table 1, Figure 1 and Figure 2).

**Measurements**

Measurements are presented in Table 1.

#### 2.1.1. Morphological Characterization

Females of *Scutellonema curcumae* sp. n. recovered from turmeric in Vietnam are characterized by a distinctly spiral to loosely coiled body upon heat relaxation (Figure 1A). The lip region is hemispherical, bearing four annuli, and is not distinctly offset from the body contour (Figure 1D,E). Basal lip annule was with 14–18 longitudinal Striations (Figure 1C). The lateral field comprises four incisures at mid-body, which become areolated in the pharyngeal region and originate approximately at the 11th body annulus, posterior to the labial region. The stylet is robust, with well-developed, rounded basal knobs (Figure 1E). The median bulb is oval and well-defined, followed by a slender isthmus encircled by the nerve ring (Figure 1E). Pharyngeal gland lobes are sacciform and overlap the intestine dorsally (Figure 1E). The secretory–excretory pore is positioned at, or slightly posterior to, the level of the pharyngeal gland terminus. A conspicuous hemizonid, approximately 1.5–2 body annuli in length, is located one annulus anterior to the secretory–excretory pore. The reproductive system is didelphic–amphidelphic, with both branches equally developed. Ovaries are outstretched, each containing a single row of oocytes arranged in tandem. The vulva is located near the mid-body; the epiptygma is distinctly folded into the vagina (Figure 1B,I,J). The spermatheca is small and empty of sperm, suggesting females are likely parthenogenetic. The tail terminus is rounded, bearing a conspicuous, rounded scutellum situated at the level of the anus; the lateral field is areolated at the level of the scutellum (Figure 1K).

Male: Not found.

#### 2.1.2. Diagnosis and Relationship

*Scutellonema curcumae* sp. n. is distinguished from congeners by the following characters: distinctly spiral to loosely coiled body after heat relaxation; hemispherical lip region with four annuli not offset from body contour; lateral field with four incisures originating near the 11th body annulus and becoming areolated anteriorly; robust stylet with rounded basal knobs; conspicuous hemizonid (1.5–2 annuli long) located one annulus anterior to the S–E pore; and a rounded tail terminus bearing a prominent scutellum with areolated lateral field at scutellar level.

Based on the dichotomous key of Kolombia et al. (2017) [6], *Scutellonema curcumae* sp. n. most closely resembles *S. magniphasma*, *S. ussuriensis*, *S. megascutatum*, and *S. sanwali*. These species can be differentiated from all other species by a non-functional spermatheca and the absence or rarity of males; a vaginal wall without dentate ornamentation; lateral field areolation at the level of the scutellum; a basal lip annulus with more than 10 longitudinal striae; and a scutellum exceeding 4 μm in diameter. Following the key of Kolombia et al. (2017) [6], *Scutellonema curcumae* sp. n. can be differentiated from *S. ussuriensis*, *S. megascutatum*, and *S. sanwali* by a longer stylet (32 (30–34) µm vs. <30 µm).

*Scutellonema curcumae* sp. n. can be differentiated from *S.* magniphasma by the lip region that is hemispherical with four annuli and bears 14–18 longitudinal striae on the basal lip annulus vs. 20–26 striae in *S. magniphasma*. The stylet of *Scutellonema curcumae* sp. n. is slightly shorter (30–34 µm), with rounded basal knobs, vs. the typically longer stylet (34–38 µm), with oval knobs and flattened anterior surfaces, in *S. magniphasma*. The hemizonid is conspicuous and positioned one annulus anterior to the secretory–excretory pore in *Scutellonema curcumae* sp. n., whereas its position in *S. magniphasma* is more variable (0–4 annuli anterior to the excretory pore). Tail morphology also differs, with *Scutellonema curcumae* sp. n. having a longer, rounded tail (14–20 µm) vs. a shorter tail in *S. magniphasma* (11.5–19 µm). Although molecular data of *S. magniphasma* are unavailable in GenBank, these combined morphological features clearly separate *Scutellonema curcumae* sp. n. from *S. magniphasma*.

*Scutellonema curcumae* sp. n. can be distinguished from *S. megascutatum* by a longer stylet (30–34 µm vs. 27.4–28.8 µm in *S. megascutatum*). The secretory–excretory pore in *Scutellonema curcumae* sp. n. is located more anteriorly compared to that of *S. megascutatum* (112–127 vs. 127–145 µm). In addition, *Scutellonema curcumae* sp. n. has a rounded tail with 8–11 annuli, while *S. megascutatum* possesses a slightly tapering tail with 9–14 annuli. In addition, *Scutellonema curcumae* sp. n. has a slightly larger scutella (5.7–7.0 µm) compared with the smaller scutella (5.0–6.5 µm) of *S. megascutatum*.

*Scutellonema curcumae* sp. n. can be distinguished from *S. ussuriensis* by a longer stylet (30–34 µm vs. 23–28.6 µm), a more slender body (a = 28–33 vs. 20–30), and a non-offset lip region compared with the slightly constricted lip region of *S. ussuriensis*. The hemizonid in *Scutellonema curcumae* lies one annulus anterior to the secretory–excretory pore, whereas it is two annuli anterior in *S. ussuriensis*. The scutellum of *Scutellonema curcumae* sp. n. is larger (5.7–7 µm vs. 4.8–6.3 µm).

*Scutellonema curcumae* sp. n. differs clearly from *S. sanwali* in having a lip region with four annuli vs. five lip annuli in *S. sanwali*. The stylet of *Scutellonema curcumae* sp. n. is longer (30–34 µm) vs. 24.5–28.0 µm in *S. sanwali*. The hemizonid in *Scutellonema curcumae* sp. n. lies one annulus anterior to the excretory pore, compared with a more anterior position (hemizonion ~10 annuli posterior to pore) in *S. sanwali*. Additionally, scutella are larger in *Scutellonema curcumae* sp. n. (typically >6 µm) vs. 4–5 µm in *S. sanwali*. These combined differences reliably distinguish *Scutellonema curcumae* sp. n. from *S. sanwali*.

Compared to species described after Kolombia et al. (2017) [6], *Scutellonema curcumae* sp. n. can be differentiated from *S. afribrachyurus* by several stable and taxonomically informative morphological traits. The hemizonid of *Scutellonema curcumae* sp. n. is located one annulus anterior to the secretory–excretory pore vs. five annuli anterior to the pore in *S. afribrachyurus*. The secretory–excretory pore in *Scutellonema curcumae* sp. n. lies at or slightly posterior to the pharyngeal gland terminus, whereas in *S. afribrachyurus* it is positioned opposite the middle to mostly the posterior part of the pharyngeal gland lobe. Tail morphology also separates the two species: *Scutellonema curcumae* sp. n. has a tail with 8–11 annuli vs. 9–17 annuli in *S. afribrachyurus*. In addition, *Scutellonema curcumae* sp. n. has a more slender body habitus (higher a-value: 28–33) compared with the broader range observed in *S. afribrachyurus* (a = 20–34). Both species share the presence of a non-functional spermatheca and the rarity of males, but their combined differences in hemizonid position, tail annulation, and body form clearly distinguish *Scutellonema curcumae* sp. n. from *S. afribrachyurus*.

*Scutellonema curcumae* sp. n. can be differentiated from *S. clavicaudatum* by the lip region (hemispherical with four distinct annuli and bearing more than ten longitudinal striae on the basal lip annulus vs. completely lacking lip annuli and instead possessing six large rectangular or trapezoidal lip blocks surrounding the labial disc). The lateral field of *Scutellonema curcumae* sp. n. is areolated at least at the level of the scutellum, contrasting sharply with the non-areolated lateral field of *S. clavicaudatum*, in which neither the anterior region nor the scutellar level shows areolation. The spermatheca of the new species is non-functional and lacks sperm, whereas *S. clavicaudatum* has a functional spermatheca that is typically filled with sperm and may range from small and rounded to large and oblong. Tail morphology further separates the two species: *Scutellonema curcumae* sp. n. has a rounded tail, 14–20 µm long with 8–11 annuli, while *S. clavicaudatum* possesses a distinctly clavate tail that widens posterior to the scutellum and bears 9–14 annuli, with apical annuli often broader. Males are absent or extremely rare in *Scutellonema curcumae* sp. n. but are present in *S. clavicaudatum*, where they exhibit a finger-like hyaline tail tip and typical reproductive features. These combined differences in lip annulation, lateral field areolation, spermathecal functionality, tail configuration, and male presence unequivocally distinguish *Scutellonema curcumae* sp. n. from *S. clavicaudatum*.

*Scutellonema curcumae* sp. n. can be differentiated from *S. tanlamense* by a larger body size (802–1062 µm vs. 643–708 µm). The stylet of *Scutellonema curcumae* sp. n. (30–34 µm) is distinctly longer than that of *S. tanlamense* (24.5–26.5 µm). The position of the secretory–excretory pore also separates the two species: in *Scutellonema curcumae* sp. n., the pore is located at or slightly posterior to the pharyngeal gland lobe terminus, whereas in *S. tanlamense* it lies 8–10 annuli posterior to the pharyngeal–intestinal junction and opposite the pharyngeal glands. The hemizonid of *Scutellonema curcumae* sp. n. is positioned one annulus anterior to the pore vs. two annuli anterior in *S. tanlamense*. The scutellum of *Scutellonema curcumae* sp. n. is larger (5.7–7 µm long) and situated at the level of the anus, whereas in *S. tanlamense* it is smaller (3.5–4 µm) and positioned three annuli posterior to the anus. *Scutellonema curcumae* sp. n. possesses a non-functional spermatheca lacking sperm, in contrast to *S. tanlamense*, which has a rounded spermatheca that may contain sperm. Combined differences in body size, stylet length, scutellum position and size, lateral field at the tail, and secretory–excretory pore position clearly distinguish *Scutellonema curcumae* sp. n. from *S. tanlamense*.

#### 2.1.3. Molecular Characterization

*Characterization of the ITS rDNA region*

Two ITS rDNA sequences of *Scutellonema curcumae* sp. n. were generated, ranging from 1219 to 1281 bp. The sequences were highly similar to one another (98%; 10 bp difference) but showed substantial divergence from all other *Scutellonema* species available in GenBank. The closest match was *S. truncatum* (DQ316098), sharing only 69–70% sequence similarity (237–244 bp difference). Bayesian phylogenetic inference placed *Scutellonema curcumae* sp. n. in a maximally supported clade (PP = 1.0) as sister to *S. truncatum*, clearly demonstrating its genetic distinctiveness (Figure 2).

*Characterization of the D2–D3 region of the 28S rDNA*

**Figure 3:**
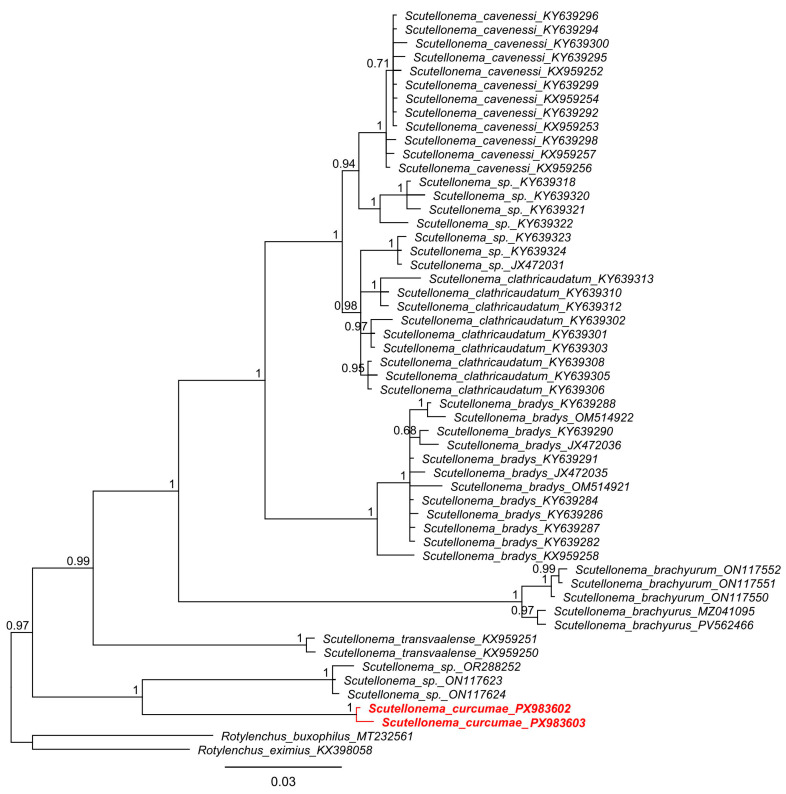
Bayesian phylogenetic tree generated using D2–D3 sequences of *Scutellonema* species under the GTR + G + I model. The sequence of *Scutellonema curcumae* sp. n. from Vietnam was indicated by bold font and red color.

Two sequences of the D2–D3 expansion segments of the 28S rDNA were obtained for *Scutellonema curcumae* sp. n., ranging from 713 to 785 bp. The sequences exhibited 99.5% similarity to each other (4 bp difference). Comparative analyses revealed high divergence from other congeners; the closest sequences were unidentified *Scutellonema* spp. (ON117623, ON117624, OR288252), with only 89.4–90.5% similarity (73–77 bp difference). In the Bayesian phylogeny, *Scutellonema curcumae* sp. n. formed a maximally supported (PP = 1.0) sister relationship with these three *Scutellonema* sp. sequences, further supporting the new species’ distinct status (Figure 3).

*Characterization of the COI mtDNA region*

**Figure 4:**
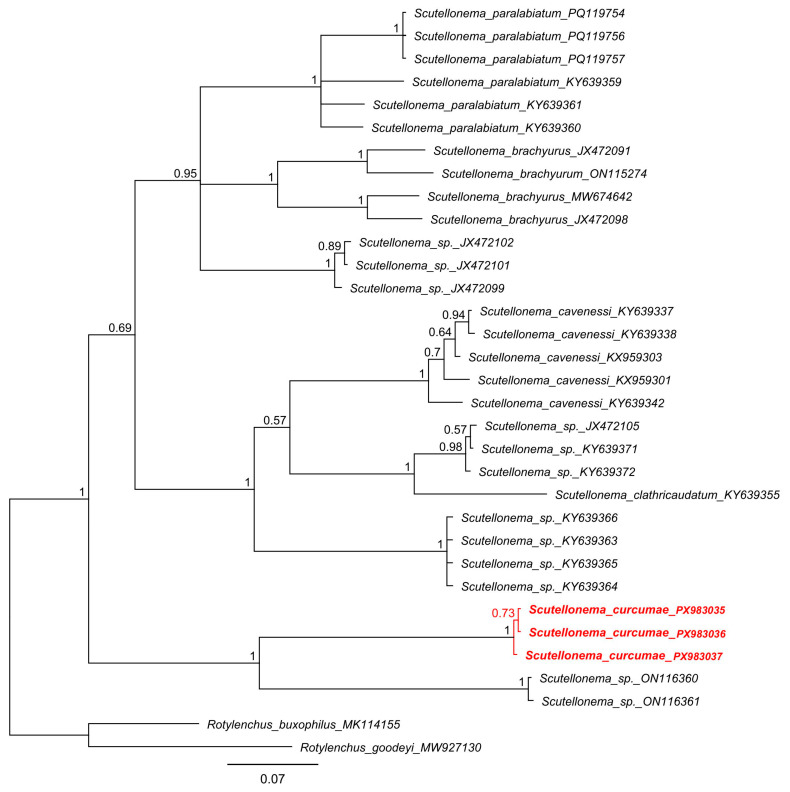
Bayesian phylogenetic tree generated using COI sequences of *Scutellonema* species under the GTR + G + I model. The sequence of *Scutellonema curcumae* sp. n. from Vietnam was indicated by bold font and red color.

Three *COI* mtDNA sequences of *Scutellonema curcumae* sp. n. were obtained, 441–443 bp in length. These sequences were nearly identical (99.8–100% similarity; 0–1 bp difference). When compared with other *Scutellonema* species, they were markedly divergent, with the closest matches being unidentified *Scutellonema* spp. (ON116360 and ON116361), sharing only 79.4–79.9% similarity (87–89 bp difference). The Bayesian tree placed *Scutellonema curcumae* sp. n. as a maximally supported (PP = 1.0) sister lineage to these *Scutellonema* sp. sequences, corroborating its distinctiveness across nuclear and mitochondrial markers (Figure 4).

*Etymology*

The specific epithet *curcumae* is derived from the generic name of the host plant, *C. longa* (turmeric), from which the new species was recovered. It is used in the genitive case, meaning “of Curcuma”.

### 2.2. Chemical Composition and Biological Activity Analysis of Turmeric in Relation to Nematode Infection

Analysis of 10 soil and turmeric rhizome samples from Kon Tum, Vietnam, revealed a clear inverse correlation between the population density of the parasitic nematode *Scutellonema curcumae* sp. n. and the biochemical quality of the rhizomes. As the density of *Scutellonema curcumae* sp. n. decreased from 19.6 (sample M1) to 0.8 (sample M10) individuals per 100 g of dry soil, the total curcuminoid content increased from 4.01% to 4.95%. This increase was observed for all three major compounds but to varying degrees: curcumin increased slightly (~8%, from 3.19% to 3.45%), demethoxycurcumin increased by ~40% (from 0.64% to 0.90%), and most notably, bisdemethoxycurcumin surged by 250% (from 0.16% to 0.56%), significantly altering the constituent ratio (Table 2).

This directly led to a marked enhancement in bioactivity. Antioxidant activity (SC_50_) improved substantially as the SC_50_ value decreased from 43.62 µg/mL to 13.85 µg/mL. Concurrently, cytotoxic activity against cancer cell lines was also potentiated, evidenced by a decrease in the IC_50_ value against HepG2 (liver) cells from 44.23 µg/mL to 29.35 µg/mL and against A549 (lung) cells from 55.23 µg/mL to 40.37 µg/mL. Notably, the two turmeric samples with the highest quality (M9, M10) were also associated with the lowest soil pH (5.9 and 5.6, respectively), suggesting that acidic soil conditions may be a suppressive factor for nematodes and/or a stimulant for curcuminoid accumulation in the plant (Table 3).

The regression analysis revealed that the root population of *S. curcumae* sp. n. had a much stronger and statistically significant negative impact on turmeric quality compared to the soil population. Specifically, bisdemethoxycurcumin was the most sensitive constituent, decreasing by 3.81% for every unit increase in root nematode density. Interestingly, the soil population showed no significant correlation with curcumin content (*p* > 0.05) (Table 4). While abiotic factors such as soil pH are known to influence secondary metabolite profiles [18], the sampled sites exhibited consistent pedological characteristics with pH values falling within the optimal range for *C. longa* (5.6–5.9). Consequently, the pronounced phytochemical degradation observed—strongly correlated with endoparasitic root burden rather than soil parameters—is attributed primarily to the direct biotic stress exerted by *Scutellonema curcumae* sp. n. feeding activity. Our results indicate that *S. curcumae* sp. n. imposes a metabolic cost similar to other major endoparasites of turmeric. For instance, Prabhu et al. [15] and Sellaperumal et al. [16] reported that high population densities of the root-knot nematode *Meloidogyne incognita* significantly reduced both rhizome biomass and curcuminoid accumulation in *Curcuma longa*. The strong negative correlation observed in our study suggests that *S. curcumae* sp. n., through its endoparasitic feeding habit, similarly disrupts root physiology and secondary metabolite synthesis, leading to the observed decline in medicinal quality.

Regarding bioactivity, root infection was identified as the primary driver of quality loss. It caused a dramatic increase in SC_50_ values (+12.33% per unit), indicating a sharp decline in antioxidant capacity. Furthermore, only the root population showed a significant negative impact on cytotoxic activity against HepG2 and A549 cancer cell lines, whereas the soil population effect was non-significant. These findings support the hypothesis that direct plant-parasitic nematode feeding damage within the rhizomes is the critical mechanism disrupting secondary metabolite accumulation and therapeutic potency.

## 3. Discussion

The present study describes *Scutellonema curcumae* sp. n., a novel spiral nematode species recovered from turmeric rhizospheres in Vietnam, through an integrative taxonomic approach combining detailed morphology and multi-locus molecular analysis (ITS, 28S D2–D3, COI). Beyond its taxonomic novelty, this investigation reveals a previously undocumented inverse correlation between the population density of this nematode and the phytochemical quality of its host.

Morphologically, *Scutellonema curcumae* sp. n. is distinguished by a unique suite of characters, including a spiral body habitus, a hemispherical lip region with four annuli, a robust stylet with rounded knobs, and a prominent, rounded scutellum. These traits align it with the *S. bradys* species complex, a group characterized by non-functional spermathecae and the rarity of males [6,19,20,21]. However, it is clearly differentiated from its closest morphological relatives, such as *S. magniphasma*, *S. ussuriensis*, *S. megascutatum*, and *S. tanlamense*, by a combination of body form, stylet length, and scutellum size and position. This morphological distinctiveness is robustly corroborated by molecular evidence. The substantial genetic divergence observed across all three markers—with sequence similarities to the closest available congeners ranging from only 69–70% (ITS) to 79–80% (COI)—falls well outside the range of typical intraspecific variation reported for *Scutellonema* species [9,22,23,24]. This strong concordance between morphological and molecular data solidifies the status of *Scutellonema curcumae* sp. n. as a distinct lineage within the genus.

While most *Scutellonema* species are not considered primary pests, several are known to cause economic damage to root and tuber crops [1]. The exclusive recovery of *Scutellonema curcumae* sp. n. from turmeric rhizospheres in this study suggests a potential host association, possibly due to an adaptation to rhizomatous plants, a trait observed in related species like *S. bradys* on yams [22,23]. This finding expands the known host range of the genus and underscores the importance of nematode surveys in medicinal crop systems, where such associations may have been previously overlooked.

The most significant finding of this work extends beyond taxonomy to plant–nematode–chemistry interactions. We demonstrate that infestation by *Scutellonema curcumae* sp. n. acts as a biotic stressor, exerting a negative impact on the biosynthesis and accumulation of curcuminoids, the principal bioactive compounds in turmeric. This aligns with and expands upon previous studies showing that nematode infection (e.g., by *Meloidogyne* spp. and *Rotylenchulus reniformis*) can reduce curcuminoid content and alter metabolite profiles in turmeric and other plants [15,16,17]. However, our study provides novel, quantitative evidence for a density-dependent effect within a single nematode species, establishing a clear gradient of phytochemical impairment directly correlated with infestation levels.

Notably, the nematode-induced stress did not uniformly suppress all curcuminoids. While total content decreased with higher infestation, the relative proportion of the three major curcuminoids shifted markedly. The most pronounced reduction was observed in bisdemethoxycurcumin, which increased by up to 250% in low-infestation samples. This differential impact suggests that the nematode may be interfering with specific enzymatic steps in the curcuminoid biosynthetic pathway, possibly those involving *O*-methylation. This hypothesis is supported by research indicating that plant defense responses to biotic stress can differentially regulate phenylpropanoid pathway genes, leading to altered profiles of secondary metabolites [24,25,26]. The preferential reduction of bisdemethoxycurcumin, the least methylated and often the most potent antioxidant in vitro among the three [22,24], implies that *Scutellonema curcumae* sp. n. infection may selectively compromise pathways yielding compounds with heightened defensive roles.

The phytochemical alterations directly translated to measurable changes in bioactivity. The enhanced antioxidant (lower SC_50_) and cytotoxic (lower IC_50_ against HepG2 and A549 cells) activities observed in rhizomes from low-infestation plots can be primarily attributed to the restored and rebalanced curcuminoid profile. This reinforces the established link between curcuminoid content and these biological activities [19,20,24]. While the observed bioactivity remains less potent than standard chemotherapeutic agents like paclitaxel, the significant variation driven by nematode pressure highlights that nematode management is a critical, yet often neglected, factor in determining the medicinal efficacy and nutraceutical value of harvested turmeric.

An intriguing secondary observation was the association between lower soil pH (5.6–5.9), lower nematode density, and higher curcuminoid content. While soil parameters (pH, moisture, N) showed limited overall fluctuation in our study, this correlation suggests that acidic soil conditions might be suboptimal for *Scutellonema curcumae* sp. n. or, conversely, might stimulate curcuminoid biosynthesis as part of a generalized stress response in turmeric. This aligns with agronomic observations that environmental stressors can enhance secondary metabolite production in medicinal plants [27,28]. However, this study was not designed to disentangle these effects, representing a clear limitation. The observed correlation warrants further controlled experiments to determine causality.

*Limitations and Future Directions:*

This study has several limitations that provide avenues for future research. First, the correlative nature of the field data, while strongly suggestive, cannot definitively establish causation between nematode density and curcuminoid profiles. Controlled inoculation experiments under greenhouse or growth chamber conditions are essential to confirm the pathogenic nature of *Scutellonema curcumae* sp. n. and its direct metabolic effects. Second, the molecular mechanisms underlying the differential suppression of curcuminoids remain unknown. Transcriptomic or metabolomic analyses of nematode-infected versus healthy turmeric roots could elucidate the specific pathways disrupted. Third, the potential interaction between soil pH and nematode virulence or plant defense requires systematic investigation. Finally, the host range and geographic distribution of *Scutellonema curcumae* sp. n. need to be determined to assess its broader agricultural significance.

In conclusion, this study achieves two key objectives: it formally describes a new nematode species, *Scutellonema curcumae* sp. n., and it provides compelling evidence that this nematode is a significant biotic stress factor that degrades the phytochemical quality of turmeric. These findings shift the perspective on nematode management in high-value medicinal crops from a sole focus on yield protection to a critical strategy for quality assurance and enhancement. By managing *Scutellonema curcumae* sp. n. populations, farmers and producers can potentially harness the plant’s stress response to cultivate turmeric with tailored, optimized curcuminoid profiles for specific medicinal or nutraceutical applications. This integrative approach, combining taxonomy, ecology, and phytochemistry, offers a refined framework for developing precision agriculture strategies aimed at maximizing both the productivity and therapeutic value of medicinal plants.

## 4. Materials, Methods, and Experiments

### 4.1. Sampling and Nematode Extraction

Soil and root samples of turmeric were collected during a nematological survey in the Western Highlands of Vietnam (exact localities provided in Section 2). Ten soil and turmeric samples were randomly collected in Kon Tum, Vietnam, at GPS coordinates 14°44′23″ N 107°74′66″ E in October 2024. The samples are deposited at the Institute of Chemistry, Vietnam Academy of Sciences and Technology.

For each field, 1 kg of soil and 100 g of roots were taken from the rhizosphere at a depth of 10–25 cm. Nematodes were extracted from soil using the modified Baermann tray method [29]. Roots were thoroughly washed under running tap water, cut into 0.5-cm fragments, and placed on modified Baermann funnels for 48 h to recover motile nematodes [29]. Extracted individuals were hand-picked under a stereomicroscope for subsequent morphological and molecular analyses.

Determination of nematode density: nematodes were counted using a counting disc. Data were processed using Microsoft Office Excel 2016 software to compute the mean.

### 4.2. Morphological Characterization

Fresh nematodes were killed by heat at 60–70 °C for 30 s and transferred immediately into TAF fixative (8 mL formalin 40%, 2 mL triethanolamine, 90 mL distilled water) following Courtney, Polley [30]. Fixed specimens were processed in anhydrous glycerin using the slow dehydration method of Seinhorst [31]. Permanent slides were prepared and sealed with paraffin. Morphometric measurements were made using an Olympus BX51 Microscope (Olympus Optical, Hamburg, Germany) equipped with an HD ultra camera. Photomicrographs and drawings were prepared for taxonomic description.

For scanning electron microscopy (SEM), specimens were prepared through a graded ethanol series (5%, 10%, 20%, 40%, 80%, 95%, and 98%), with specimens immersed for 20 min at each concentration, followed by three additional 10-min changes in absolute ethanol. The material was then subjected to critical-point drying using liquid CO_2_ as the transitional medium. Dried specimens were mounted on aluminum stubs with double-sided conductive carbon adhesive tabs and sputter-coated with a 10 nm layer of gold (120 s at 15 mA). Observations and image acquisition were performed using a JSM-840 scanning electron microscope (JEOL Ltd., Akishima, Japan) operated at an accelerating voltage of 12 kV following Abolafia (2015) [32].

### 4.3. Molecular Characterization

Live nematodes were isolated and washed three times in sterile distilled water. Individual specimens were transferred to PCR tubes and cut into small fragments in 20 µL worm lysis buffer (50 mM KCl, 10 mM Tris-HCl, pH 8.3, 2.5 mM MgCl_2_, 0.45% NP-40, 0.45% Tween-20). Samples were incubated at −20 °C for at least 10 min before adding 1 µL proteinase K (1.2 mg/mL). Lysis was performed at 65 °C for 1 h, followed by enzyme inactivation at 95 °C for 10 min.

The primers D2A/D3B were used to amplify the D2-D3 expansion segments of the 28S rRNA [32]. PCR amplification began with an initial denaturation at 94 °C for 4 min, followed by five cycles of 94 °C for 30 s, 45 °C for 30 s, and 72 °C for 2 min. This was followed by 35 cycles of 94 °C for 30 s, 54 °C for 30 s, and 72 °C for 1 min, and the reaction was finalized at 12 °C for 10 min. The ITS rDNA region was amplified using primers Vrain2F/Vrain2R [33,34], with PCR conditions consisting of an initial denaturation at 94 °C for 4 min followed by 50 cycles of 94 °C for 30 s, 54 °C for 30 s, and 72 °C for 2 min. The mitochondrial cytochrome c oxidase subunit I (COI) gene fragment was amplified using primers JB3/JB4 [35]. To amplify the COI mtDNA gene, the same thermal profile was used, with an annealing temperature of 45 °C. All PCR products were examined by agarose gel electrophoresis, and successful amplifications were purified and sequenced commercially by Macrogen Inc. (Europe, Amsterdam, The Netherlands).

Raw chromatograms were assembled and edited in Geneious R11 (Biomatters Ltd., Auckland, New Zealand). BLAST+ 2.16.0 searches were used to evaluate similarity to existing sequences in GenBank. Sequences were aligned using MUSCLE v5.3, and phylogenetic analyses were conducted in MrBayes v3.2.6. Best-fit nucleotide substitution models were selected using MEGA7 v7.0 based on the Akaike Information Criterion (AIC).

### 4.4. Chemical Composition and Biological Activity Analysis of Turmeric in Relation to Nematode Infection

This study investigated the influence of the nematode *Scutellonema curcumae* sp. n. on several key parameters: overall soil characteristics (pH, moisture content, and total nitrogen), turmeric plant metrics, curcuminoid content, and specifically the concentrations of curcumin, demethoxycurcumin, and bisdemethoxycurcumin. The analytical methods employed were as follows:

Soil pH measurement was conducted in accordance with the Vietnam TCVN 5979:2021 (ISO 10390:2021) standard [36].

Drying method (Gravimetric method), following the ref. [37].

Kjeldahl method (Vietnam TCVN 6498:1999): This is a standard method for determining nitrogen in most forms (except nitrate) [38].

Quantification of (High-Performance Liquid Chromatography): Quantitative analyses were performed using dried turmeric rhizomes (210 days old) collected. HPLC analysis was conducted using an Agilent 1260 model (Santa Clara, CA, USA) with a UV detector. An HPLC Eclipse XDB-C18 column (150 × 4.6 mm, 5 μm) and a guard column (pre-column) of the same XDB-C18 type from Agilent were used for chromatographic separation. The mobile phase consisted of a solvent system with channel A: H_2_O + 0.1% FA—channel B: ACN—channel C: MeOH, run isocratically at a 40–25–35 ratio for 30 min. Flow rate: 0.5 mL/min; Injection volume (auto-sampler): 5 µL; Total sample analysis time: 30 min; Column temperature: 30 °C; Flow pressure: 64 bar; UV/Vis detector: Set at a wavelength of 428 nm [39,40].

*In vitro antioxidant activity assay*: Analysis of the ability to scavenge free radicals generated by DPPH (1,1-diphenyl-2-picrylhydrazyl) is a recognized method for rapid determination of antioxidant activity. The test substance was dissolved in 100% dimethyl sulfoxide (DMSO), and DPPH was prepared in 96% ethanol. The absorbance of DPPH at λ = 515 nm was determined after adding DPPH to the test solution on a 96-well microplate. Test results are expressed as the mean of at least three replicate trials ± standard deviation. Samples were prepared in 100% DMSO at 4 mg/mL for crude extracts and 1 mg/mL for purified samples. A 5 mM ascorbic acid in 10% DMSO was used as a positive control. Samples were pipetted onto a 96-well microplate with DPPH solution to achieve final test concentrations in the reaction ranging from 200 μg/mL to 12.5 μg/mL. Incubation was at 37 °C for 30 min, and optical density (OD) was measured at λ = 515 nm using a photometer (Infinite F50, Tecan, Männedorf, Switzerland). The test substance was diluted into decreasing concentrations, with three replicates at each concentration. The SC_50_ value (μg/mL), the concentration of the test substance that neutralizes 50% of free radicals, was determined using TableCurve v5.0 AISN Software (Jandel Scientific, San Rafael, CA, USA) based on the SC% value (free radical scavenging capacity) and the corresponding range of test concentrations [41,42].

*In vitro MTT assay*: MTT [3-(4,5-dimethylthiazol-2-yl)-2,5-diphenyltetrazolium bromide] was evaluated by the US National Cancer Institute (NCI) as a standardized, effective method for rapid screening of substances with cytotoxic or cell-proliferation-inhibitory activity. The principle of the method is the indirect determination of test substance activity through its ability to inhibit NAD(P)H-dependent oxidoreductase enzymes in cells. This mitochondrial enzyme catalyzes the reduction of the tetrazolium dye MTT to an insoluble purple formazan product, which can reflect the relative number of viable cells when measured at λ = 540/720 nm. Cell lines: Hep-G2 (Hepatocellular carcinoma), A549 (Human lung adenocarcinoma epithelial cells), provided by ATCC (American Type Culture Collection, Manassas, VA, USA; https://www.atcc.org). Cells were cultured at 37 °C, 5% CO_2_ in suitable media: DMEM (Dulbecco’s Modified Eagle Medium), EMEM (Eagle’s Minimum Essential Medium, Sigma-Aldrich, St. Louis, MO, USA), or RPMI 1640 (ThermoFisher, Waltham, MA, USA) supplemented with 2 mM L-glutamine, antibiotics (Penicillin + Streptomycin sulfate), and 5–10% fetal bovine serum. The cell suspension was then pipetted onto a 96-well microplate (1.5 × 10^5^ cells/well) and incubated with test samples at concentration ranges from 100–6.25 µg/mL for crude extracts or 50–1 µg/mL (µM) for purified compounds, with each concentration repeated three times. Paclitaxel (Taxol) in DMSO was used as a positive control (+). The metabolized crystalline formazan product was dissolved in dimethyl sulfoxide (DMSO, Sigma-Aldrich), and optical density was measured at λ = 540/720 nm using an Infinite F50 instrument (Tecan, Männedorf, Switzerland). Samples showing activity (% inhibition ≥ 50%) had their IC_50_ value (µg/mL) determined, which is the concentration of the test sample that inhibits 50% of cell survival, using TableCurve AISN Software (Jandel Scientific, San Rafael, CA, USA) [42,43].

## 5. Conclusions

This study successfully integrates nematode taxonomy and phytochemical analysis to reveal a new species and its significant impact on a key medicinal crop. We formally describe *Scutellonema curcumae* sp. n., a novel spiral nematode recovered from turmeric rhizospheres in Vietnam, distinguished by unique morphological features and confirmed by substantial molecular divergence. This represents the first report of a *Scutellonema* species associated with turmeric in Vietnam, expanding the known biodiversity and potential pest spectrum for this valuable crop.

Beyond taxonomy, our findings establish that *Scutellonema curcumae* sp. n. infestation directly correlates with a deterioration in turmeric quality. Higher nematode densities lead to reduced total curcuminoid content and a disproportionate decrease in bisdemethoxycurcumin, resulting in diminished in vitro antioxidant and cytotoxic activities. This positions nematode management not merely as a yield-protection strategy but as an essential practice for safeguarding and enhancing the medicinal value of turmeric.

To translate these findings into practical applications, future research should focus on: (i) confirming pathogenicity through controlled inoculation trials; (ii) elucidating the molecular mechanisms behind the differential curcuminoid suppression using transcriptomic/metabolomic approaches; and (iii) investigating the interaction between soil properties and nematode impact to develop integrated management strategies.

In summary, this work identifies *Scutellonema curcumae* sp. n. as both a taxonomic novelty and a biotic stressor that compromises turmeric quality. Our findings provide a critical foundation for developing precision agriculture practices aimed at optimizing the phytochemical profiles and therapeutic potential of medicinal plants.

## Figures and Tables

**Table 1:** Measurements of females of *Scutellonema curcumae* sp. n. from Vietnam and closely resembled species. All measurements are in μm (except for ratio) and in the form: mean ± s.d. (range).

	*Scutellonema curcumae* sp. n.		*S. magniphasma*	*S. ussuriensis*	*S. megascutatum*	*S. sanwali*
	Holottype	Paratypes				
n	1	10	10	11	20	12
L	917	922 ± 81 (802–1062)	891 (820–980)	0.77 (0.63–0.98)	870 ± 57 (790–990)	730 ± 56 (620–880)
V%	56	55 ± 0.5 (54–55)	56 (55–61)	-	58 ± 32.8 (56–64)	59 ± 1 (57–61)
G1	202	191 ± 16 (169–223)	-	-	-	-
G2	198	190 ± 17 (166–225)	-	-	-	-
Lip width	10.5	10.7 ± 0.6 (9.8–11.6)	-	-	9.5 (7.3–10.1)	-
Lip height	5.2	5.4 ± 0.4 (4.7–6.1)	-	-	5.2 (4.8–6.1)	-
Sylet length	32	32 ± 1.3 (30–34)	31 (29–35)	25.2 (23.4–28.6)	28.1 ± 0.44 (27.4–28.8)	25 ± 0.9 (25–28)
cone	13.7	13.6 ± 0.8 (12.5–15)	-	-	13.7–14.6	-
shaft	13.1	13.9 ± 0.7 (12.6–15)	-	-		-
knob	4.7	4.6 ± 0.3 (4.2–5.2)	-	-	2.4–3.9	-
DGO	7.8	7.1 ± 1.1 (5.2–9)	-	-	7.8 (6–9)	-
Max body diam. (MBD)	31	31 ± 3 (27–35)	-	-	29 (27–33)	-
Anterior end to secretory-excretory pore (EP)	121	120 ± 4 (112–127)	-	-	134 (127–145)	-
Anterior end to nerve ring	101	99 ± 5.3 (89–105)	-	-		-
ant-junction intestin	106	111 ± 6 (101–123)	-	-		-
Pharynx overlapping	35	25 ± 6 (17.5–35)	-	-	14–34	-
Vulval body diam. (VBD)	31	32 ± 3 (28–35)	-	-		-
Ana body diam. (ABD)	26	27 ± 3 (23–32)	-	-		-
Tail length	17.2	17.5 ± 1.9 (14–20)	-	-	12.8 ± 1.9 (10.3–16.4)	-
scutellum length	6.6	6.4 ± 0.4 (5.7–7)	-	-		-
scutellum width	5.8	5.5 ± 0.4 (4.7–5.9)	6.5 (5–7)	4.8–6.3	5.9 ± 0.43 (5.0–6.5)	4–5
Tail annuli	11	10.2 ± 1.2 (8–11)	-	-	-	-
a = L/MBD	29	30 ± 2 (28–33)	27 (24–30)	24.5 (20–30)	30 ± 2.62 (25–34)	29 ± 2 (28–32)
b = L/ES	8.7	8.3 ± 0.6 (7.5–9.3)	7.3 (7–8)	6.6 (5.2–9.3)	7.8 ± 0.72 (6.5–9.0)	6.3 ± 0.6 (8.8–7.4)
c = L/T	53	53 ± 4.3 (46–62)	53 (48–75)	53 (45–71)	71 ± 13.5 (54–96)	76 ± 2 (68–88)
c’ = T/ABD	0.65	0.66 ± 0.05 (0.57–0.74)	0.57 (0.5–0.8)	-	0.7 ± 0.1 (0.5–0.8)	-

**Table 2:** Soil physicochemical properties, soil type, and nematode population density in *Curcuma longa* cultivation sites in Kon Tum, Vietnam.

Samples	Soil Type ^a^	pH	Moisture (%)	Total Nitrogen (%)	Total Nematodes (Soil) ^b^	Total Nematodes (Root) ^b^	*Scutellonema curcumae* sp. n. (Soil) ^b^	*Scutellonema curcumae* sp. n. (Root) ^b^
M1	Reddish-brown basaltic	6.5	73.5	1.05	131.8	59.7	25.3	19.6
M2	Reddish-brown basaltic	6.3	73.4	0.92	58.2	17.7	11.4	7.5
M3	Reddish-brown basaltic	6.6	73.6	0.95	79.3	88.4	3.8	10.7
M4	Reddish-brown basaltic	7.1	74.1	0.98	185.3	78.4	27.9	12.5
M5	Reddish-brown basaltic	6.8	74.2	1.07	125.3	58.4	29.6	16.5
M6	Reddish-brown basaltic	6.9	74.0	1.01	141.5	66.8	22.1	12.3
M7	Reddish-brown basaltic	6.0	72.8	0.90	87.6	33.9	5.5	8.2
M8	Reddish-brown basaltic	6.2	72.9	0.91	90.2	47.9	6.3	3.1
M9	Reddish-brown basaltic	5.9	72.8	0.95	70.1	23.3	4.5	1.6
M10	Reddish-brown basaltic	5.6	70.9	0.92	36.8	11.3	2.6	0.8

^a^ Soil texture was visually assessed as clay loam, typical of the regional Ferralsols (Red Basalt soil). ^b^ Individuals per 100 g of soil/root.

**Table 3:** Phytochemical composition and biological activities of *Curcuma longa* rhizomes in relation to nematode infection.

Samples	Total Curcuminoids (%)	Curcumin (%)	Demethoxy Curcumin (%)	Bisdemethoxy Curcumin (%)	SC_50_ (μg/mL)	IC_50_ HepG2 (μg/mL)	IC_50_ A549 (μg/mL)
M1	4.01 ± 0.05	3.19 ± 0.08	0.64 ± 0.04	0.16 ± 0.01	43.62 ± 3.08	44.23 ± 2.08	55.23 ± 3.34
M2	4.25 ± 0.03	3.07 ± 0.07	0.86 ± 0.02	0.32 ± 0.03	23.45 ± 2.19	43.67 ± 2.86	58.93 ± 2.99
M3	4.16 ± 0.04	3.06 ± 0.05	0.80 ± 0.03	0.31 ± 0.02	21.03 ± 2.56	45.37 ± 3.49	60.32 ± 3.05
M4	4.15 ± 0.02	3.18 ± 0.05	0.72 ± 0.02	0.23 ± 0.01	32.89 ± 3.55	38.82 ± 2.98	52.75 ± 2.45
M5	4.10 ± 0.04	3.19 ± 0.06	0.69 ± 0.04	0.21 ± 0.02	40.31 ± 3.86	37.85 ± 2.05	50.37 ± 2.97
M6	4.15 ± 0.05	3.21 ± 0.06	0.69 ± 0.01	0.23 ± 0.01	34.26 ± 3.82	38.63 ± 2.52	51.34 ± 3.14
M7	4.23 ± 0.03	3.16 ± 0.09	0.74 ± 0.02	0.31 ± 0.03	25.87 ± 2.44	39.38 ± 1.56	48.21 ± 3.82
M8	4.70 ± 0.06	3.38 ± 0.07	0.91 ± 0.03	0.41 ± 0.02	18.91 ± 1.73	35.76 ± 2.88	47.55 ± 2.43
M9	4.88 ± 0.07	3.45 ± 0.08	0.98 ± 0.04	0.46 ± 0.01	16.42 ± 1.89	29.04 ± 1.22	41.43 ± 3.02
M10	4.95 ± 0.02	3.45 ± 0.04	0.90 ± 0.02	0.56 ± 0.02	13.85 ± 1.31	29.35 ± 1.10	40.37 ± 3.31
Ascorbic acid	-	-	-	-	6.81 ± 1.03	-	-
Paclitaxel	-	-	-	-	-	47.2 ± 1.38 nM	21.3 ± 1.02 nM

**Table 4:** Linear regression analysis of *Scutellonema curcumae* sp. n. population density (Root vs. Soil) on chemical composition and biological activity.

Target Parameter	Root Population Impact (% Change per Unit)	Soil Population Impact (% Change per Unit)	Combined Impact
**Chemical constituent**			
Total curcuminoids	−1.04	−0.46	−0.35
curcumin	−0.44	NS	NS
Demethoxycurcumin	−1.78	−0.89	−0.64
Bisdemethoxycurcumin	−3.81	−2.05	−1.41
**Biological Activity**			
Antioxidant activity (SC_50_)	+12.33	+5.5	+4.51
Cytotoxicity on HepG2 (Liver cancer, IC_50_)	+1.94	NS	NS
Cytotoxicity on A549 (Lung cancer, IC_50_)	+1.47	NS	NS

NS = Not significant (*p* > 0.05). Positive values in SC_50_/IC_50_ indicate reduced potency.

## Data Availability

The original contributions presented in this study are included in the article. Further inquiries can be directed to the corresponding authors.
